# Warming and acidification alter essential fatty acid profiles in marine diatom *Skeletonema marinoi*

**DOI:** 10.1093/plankt/fbag057

**Published:** 2026-07-03

**Authors:** Maria Nicoară, Charlotte L Briddon, Alexandra Mortu, Adriana Hegedűs, Emese Gal, Horia Leonard Banciu, Bogdan Drugă

**Affiliations:** Institute of Biological Research Cluj, National Institute of Research and Development for Biological Sciences, 48 Republicii Street, Cluj-Napoca 400015, Romania; Doctoral School of Integrative Biology, Faculty of Biology and Geology, Babeș-Bolyai University, 44 Republicii Street, Cluj-Napoca 400015, Romania; Institute of Biological Research Cluj, National Institute of Research and Development for Biological Sciences, 48 Republicii Street, Cluj-Napoca 400015, Romania; School of Geographical Sciences, University of Bristol, University Road, Clifton, Bristol BS8 1SS, UK; Faculty of Life Sciences, University of Vienna, 1 Djesrassiplatz, 1030 Vienna, Austria; Institute of Biological Research Cluj, National Institute of Research and Development for Biological Sciences, 48 Republicii Street, Cluj-Napoca 400015, Romania; Department of Chemistry, Babeș-Bolyai University, 11 Arany Janos, Cluj-Napoca 400028, Romania; Department of Molecular Biology and Biotechnology, Faculty of Biology and Geology, Babeș-Bolyai University, 44 Republicii Street, Cluj-Napoca 400015, Romania; Centre for Systems Biology, Biodiversity and Bioresources, Faculty of Biology and Geology, Babeș-Bolyai University, 44 Republicii Street, Cluj-Napoca 400015, Romania; Institute of Biological Research Cluj, National Institute of Research and Development for Biological Sciences, 48 Republicii Street, Cluj-Napoca 400015, Romania

**Keywords:** Diatoms, polyunsaturated fatty acids, climate change, warming, acidification

## Abstract

Marine diatoms are key components of the planetary ocean, playing crucial roles in trophic networks: fixing carbon, producing fatty acids (FA) that cannot be synthesized *de novo* by higher trophic organisms. To better comprehend how the combined action of climatic change influences the dietary value and the impacts on the upper trophic levels, we used a 2 × 2 factorial experiment to investigate how the FA profiles of *Skeletonema marinoi* changed in response to the individual and combined effects of warming (7, 19°C) and acidification (400, 1 000 ppm CO_2_). Three *S. marinoi* strains were exposed for ~ 40 weeks to ambient conditions, warming, acidification and their combination and analyzed for FA profiles, focusing on polyunsaturated (PUFA), omega-3 and omega-6 FA. We found that omega-3 FA increased under warming exposure, while acidification alone led to lower omega-3/omega-6 ratios. In contrast, the ratios increased under warming, alone or coupled with acidification, in all three strains, indicating better food quality for higher trophic levels. Our results suggest the long-term exposure to both drivers will help the marine diatoms to acclimatize to their combined effects, allowing them to buffer the changes brought by warming and ocean acidification.

## INTRODUCTION

Global change triggered by anthropogenic drivers is predicted to have a detrimental impact on the marine food web (Rossoll *et al.*, 2012; Priya *et al.*, 2023). Approximately 25% of anthropogenic CO_2_ emissions have been absorbed by the oceans, altering the carbonate chemistry in a process known as ocean acidification (OA) (Devi and Mathew, 2024) leading to a reduction in the surface ocean pH by c. 0.1 units since pre-industrial times (Falkenberg *et al.*, 2020; Shajedul, 2023). The IPCC scenario (2023) predicts that, under the “business as usual” scenario with emissions remaining unchecked, future atmospheric CO_2_ concentrations could increase from current level of ~ 400 to ~ 1 000 μatm (ppm), while sea surface is predicted to further increase with 3°C by the end of the century (IPCC, 2023). Previous studies have shown that both enhanced temperature and OA affect organisms through reduced production of fatty acids (hereinafter FA), including saturated (SFA), monounsaturated (MUFA), and polyunsaturated FA (PUFA; Tseng *et al.*, 2021). Two classes of PUFA, namely omega-3 (n-3) and omega-6 (n-6), are also known for the multiple benefits on human health (Lee *et al.*, 2009), while a balanced uptake of n-3 and n-6 PUFA is recommended for protection against metabolic disease and proper brain function (Bazinet and Layé, 2014; Saini and Keum, 2018).

PUFA are not synthesized *de novo* by heterotrophic organisms, which must acquire them via their diet of phytoplankton (Bermúdez *et al.*, 2016). Warming and OA can alter the nutritional quality of marine phytoplankton as a food source for organisms higher up the trophic food web, as enhanced *p*CO_2_ can stimulate carbon fixation and possibly alter phytoplankton cellular stoichiometry (Meyers *et al.*, 2019; Van de Waal *et al.*, 2010). OA and temperature can decrease the essential FA content of microalgae and the ratio of PUFA:FA (Rossoll *et al.*, 2012), yet some studies have determined that OA can have a limited influence on PUFA synthesis (King et al., 2015). As primary producers, microalgae are the base of the marine food webs and understanding how climatic change will impact on their quality as food for the higher trophic levels is of the upmost importance.

*Skeletonema marinoi* is a dominant bloom-forming diatom and crucial primary producer in temperate coastal waters (Johansson *et al.*, 2019). It synthesizes essential omega-3 and omega-6 FA (Cardoso *et al.*, 2020), with some of the most relevant PUFA being omega-3: alpha-linolenic acid (ALA, C18:3n-3), docosahexaenoic acid (DHA, C22:6n-3), eicosapentaenoic acid (EPA, C20:5n-3), omega-6: linoleic acid (LA, C18:2n-6), and arachidonic acid (ARA, C20:4n-6). Wu *et al.* (2022) have shown that elevated CO_2_ increased MUFA and PUFA, but reduced SFA in *S. marinoi*. In contrast, other studies have found that enhanced CO_2_ leads either to no change in the PUFA content in diatoms (King *et al.*, 2015) or to their decline (Leu *et al.*, 2013). Nevertheless, the variation of PUFA concentration under different temperatures in *Skeletonema* sp*.* remains unclear and the combined effect of temperature and OA on PUFA is unknown. Therefore, analyzing the FA composition after a long-term adaptation process allows for the understanding of how *S. marinoi* would respond over time to the ongoing global climate change.

The aim of this study was to understand how experimentally-induced long-term adaptation to high temperatures and CO_2_ concentrations influences the FA profiles of three *S. marinoi* strains isolated from the coasts of Norway. In a 2 × 2 factorial design, *S. marinoi* strains were cultured for ~40 weeks under two temperatures (7 and 19°C) and two CO₂ regimes (400 ppm and 1000 ppm), and their FA profiles were analyzed. Our first hypothesis was that warming would decrease the abundance of PUFA relative to the total FA, based on the homeoviscous adaptation theory (Guschina and Harwood 2006). We also hypothesized that the singular or combined action of the two drivers—temperature and CO_2_—would decrease the omega-3 to omega-6 ratio in *S. marinoi* strains, with potential impacts on the food quality of this species for aquatic organisms higher up the trophic chain (Hixson and Arts, 2016).

## MATERIALS AND METHODS

### *Skeletonema marinoi* strains: isolation and cultivation

The *S. marinoi* strains were collected from the North and South of the Norwegian Coast using an automatic sampling system operated by NIVA (Bergen, Norway) on the coastal steamer *MS Trollfjord*. Details on the sampling, isolation and taxonomic affiliation for S8 and S16 strains were discussed in (Briddon *et al.*, 2025). We followed the same procedure for the strain S17, which was collected from Sognesjøen region (61.1554° N, 6.5806° E). The phylogenetic identity was confirmed via sequencing of 18S rDNA gene (Macrogen Europe, Amsterdam, The Netherlands). The sequences have been deposited in GenBank under the accession numbers PP600221 (S8), PP600222 (S16) and PX444878 (S17).

The strains were grown in triplicate in 100 mL glass tubes in artificial seawater (see (Briddon *et al.*, 2023)) and immersed in heated aquaria according to the experimental conditions. Cultures were bubbled for 15 min every 3 h (total 120 min daily), with filtered atmospheric air (0.22 μm; Minisart, Sartorius, Gottingen, Germany) for the ambient conditions (pH 8.13–8.17, 400 ppm CO_2_), or with artificial air containing 0.1% CO_2_ for the OA scenarios (pH 7.77–7.82, 1 000 ppm; Messer, Bad Soden, Germany). Both pH values are on National Bureau of Standards (NBS) scale. Light was provided by LED lamps (100 μmol photon m^−2^ s^−1^) under light:dark cycles of 16:8 h. Alkalinity has been kept constant between 2290 and 2310.

### Experimental design and sample processing

**Fig. 1:**
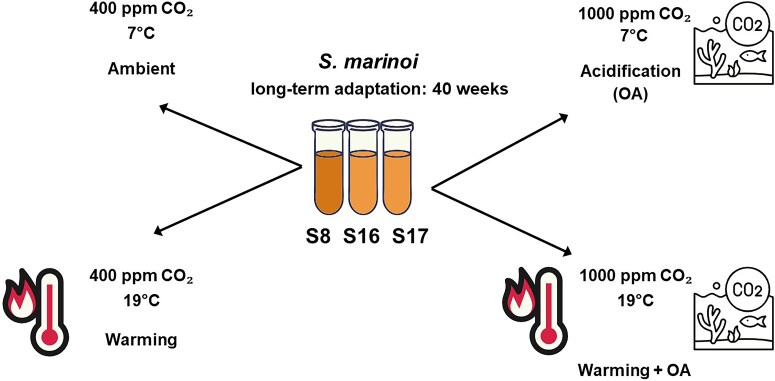
Experimental design: 2 × 2 factorial experiment: 2 factors (temperature and CO_2_) with 2 levels each (7°C, 19°C and 400 ppm, 1000 ppm). Created using canva.com

The individual and combined effects of temperature and CO_2_ have been investigated in four scenarios (Fig. 1): 1. ambient (7°C × 400 ppm); 2. warming (19°C × 400 ppm CO_2_); 3. acidification (7°C × 1000 ppm) and 4. warming coupled with acidification (19°C × 1000 ppm). These scenarios aimed at serving as proof of concept for the effects of global change and bring empirical evidence on the impact of extreme climate conditions on key phytoplankton species. The long-term exposure to temperature (7 and 19°C) lasted from August 2021 until November 2022 (15 months), while for CO_2_ (400 and 1000 ppm), it lasted 12 months (November 2021–2022). These conditions were chosen to reflect the scenarios for the Sea of Norway for SSP5–8.5 from the climate model output (CMIP6; (IPCC, 2018)).

The cultures were regularly checked for contaminants under the microscope throughout the adaptation process. They have been diluted every 3 weeks for 19°C trial and every 5 weeks for the 7°C condition to account for the temperature-dependent influence on the growth rates (Sherman *et al.*, 2016). Accordingly, the experimental starting point was designated as weeks 1 through 6, whereas the ending point was defined as weeks 39 through 43. After every dilution, the biomass from the three replicates was centrifuged, washed with 3% NaCl solution, and stored at −20°C until FA analysis. There was no biomass analyzed in December 2021 due to sample loss.

### Analysis of FA profiles

Fatty acid methyl esters (FAME) were prepared from 45 to 100 μg of dried *S. marinoi* (lyophilized) biomass, adding 750 μL of methanolic solution with acetyl chloride (20:1). After 3 h of reaction, 500 μL of water and hexane were added to the reaction mixture and ultrasonic bath was applied to perform the FAME extraction. The organic phase was collected and 2 μL of the hexane solution was submitted to Gas Chromatography–Mass Spectrometry (GC–MS) analysis on a GC system (Shimadzu 2010, Kyoto, Japan) equipped with a ZB-5MS Plus column with the following specification: 30 m ×0.25 mm ×0.25 μm (Phenomenex, Torrance, CA, USA). The GC equipment was connected to a Shimadzu QP 2010 PLUS quadrupole detector, operating with an electron impact mode at 70 eV, scanning the range between m/z 30–600 at full scan mode acquisition. The oven temperature was programmed from the initial temperature of 60°C for 3 min, a linear increase to 120°C at 30°C min^−1^ for 5 min, then at 5°C min^−1^ to 250°C, and then at 20°C min^−1^ to 300°C, standing for 10 min. The injector and detector temperatures were 300 and 220°C, respectively. Helium (99.99990% purity Linde) was used as the carrier gas at a flow rate of 0.85 mL min^−1^. The injection volume was 2 μL, made in split mode (20:1) at 300°C. The compound identification was performed by MS spectrum comparison with the database (NIST 27, 147 and Wiley library). Identification of FAME was performed by comparing their retention times and MS spectra with those obtained from the FAME standard mixture (Supelco® 37 Component FAME Mix). No co-elution tests or internal standards were applied. The relative percentage of each FA was estimated as a fraction of its integrated ion area from the total ion chromatogram area (100%).

### Data analysis

The triplicate cultures were mixed during centrifugation and FAME analysis was conducted, for each strain, on the combined biomass. The resulting gas chromatograms were analyzed after excluding the unidentified compounds. The relative abundance (SFA%, MUFA%, PUFA%) was calculated as the sum of every FA, according to their classification, out of the total FA (100%, see Supplementary S1). For every experimental condition, the change in abundance of each PUFA was calculated by subtracting the initial abundance from the final abundance (end—start, Supplementary S2). Data was tested for normality (Shapiro–Wilk test) and analyzed using parametric or non-parametric tests according to their distribution. The individual and combined effects of temperature and CO_2_ on omega-3/omega-6 ratios (n-3/n-6) across time were determined as a function of strain, temperature, CO_2_ by using ANOVA on log-transformed data to ensure normality. The best fitting model was chosen based on AIC selection (Akaike Information Criterion) (Supplementary S3). Results were considered statistically significant for *P* < 0.05 and confirmed after pairwise multiple comparisons. Data analysis and visualization were conducted using R “stat”, “car” and “ggplot2” packages (version 4.4.0). Combining the three replicated cultures was necessary due to logistical restrains and in order to obtain sufficient biomass for FA analysis. However, we acknowledge this limitation lowered the statistical power of the results.

## RESULTS

### Strain-specific alterations in the FA profiles of *S. marinoi*

**Fig. 2:**
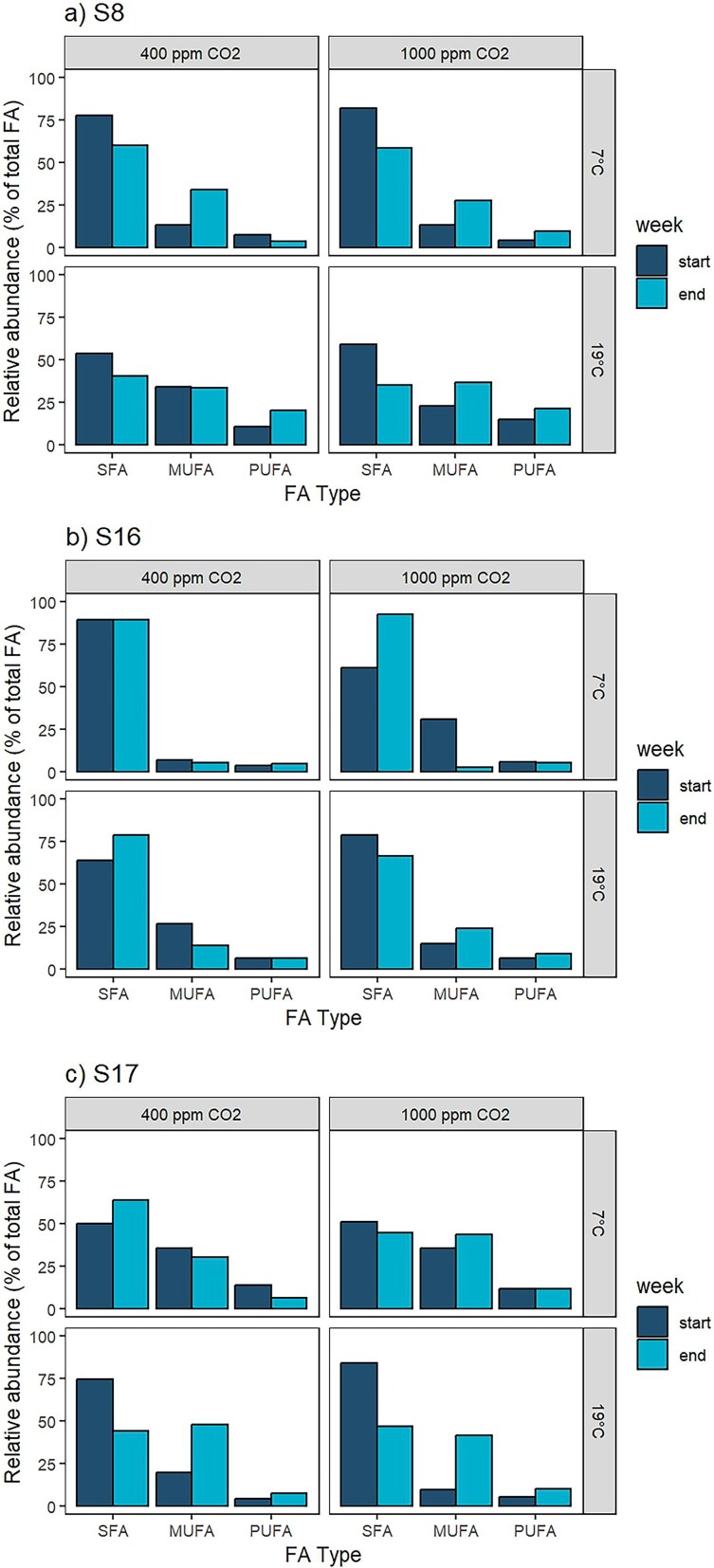
Bar plots displaying the abundance of FA types (% of total FA; saturated—SFA, monounsaturated—MUFA, polyunsaturated— PUFA) for strains a) S8, b) S16 and c) S17 after the long-term exposure to control (7°C × 400 ppm), acidification (7°C × 1000 ppm), warming (19°C × 400 ppm) and both drivers (19°C × 1000 ppm).

In strains S8 and S17, SFA abundance decreased after the exposure to warming, acidification and combined (Fig. 2), while in S16, only the combined action of the drivers reduced SFA% (−12.22% of total FA). SFA abundance increased only for S16, under the individual action of warming (+14.86%) and acidification (+31.07%).

Warming alone increased PUFA levels in S8 (+9.17%) and S17 (+3.67%) when expressed as a proportion of total FA, with no effect on S16 (+0.01%). However, when considering the PUFA pool alone, these changes were more pronounced, with PUFA substantially increasing in S8 (+85%) and S17 (+95%). Acidification, on the other hand, increased PUFA% only in S8 (+5.36% of total FA and + 135% in the PUFA pool), having no effect on S17 (+0.05%) and leading to lower PUFA% in S16 (−0.68%). PUFA levels increased in all strains after ~40 weeks of exposure to both drivers combined (+6.55% in S8, +2.45% in S16, +4.95% in S17). When considering the PUFA pool alone, these changes were more pronounced, corresponding to an increase of +45% in S8, +39% in S16, and an approximate doubling in S17 (+99%). A similar trend was observed for MUFA abundance in the three strains, which displayed higher values at the end of the exposure to warming and acidification than in the beginning (+13.67% in S8, +9.13% in S16, +28.8% in S17). These changes correspond to substantially larger increases within the MUFA fraction itself, of ~50% in S8 and S16, and ~300% in S17.

**Fig. 3:**
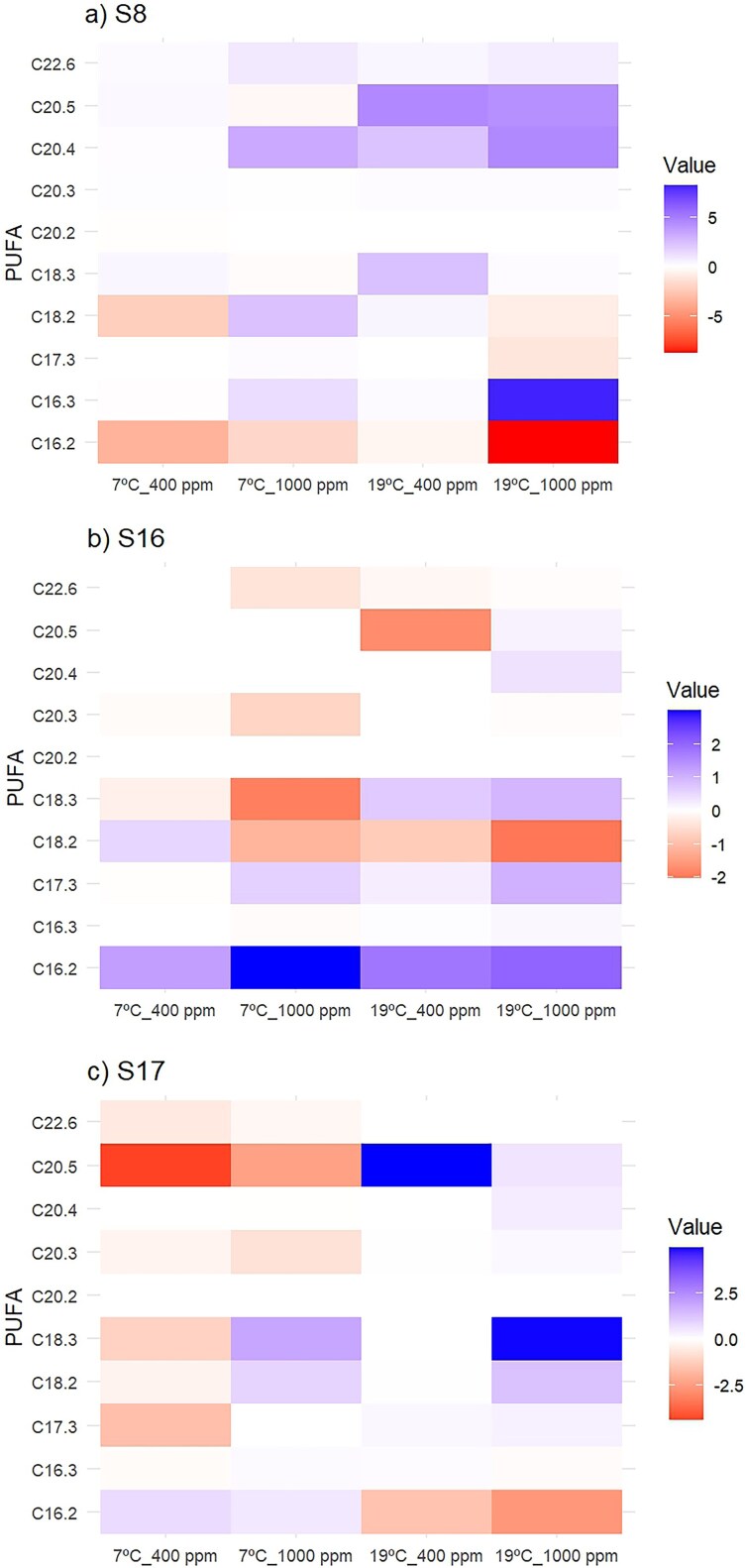
Heatmaps showing the change of abundance in specific PUFA for strains a) S8, b) S16 and c) S17. The values represent the difference between the final and initial abundance of each PUFA (end-start, % of total FA). All variants of each FA were considered, regardless of the position of the double bonds. C16:2—hexadecadienoic acid, C16:3—hexadecatrienoic, C17:3—heptadecatrienoic acid, C18:2—octadecadienoic acid (linolenic—LA and trans-linolenic—Trans-LA), C18:3—octadecatrienoic acid (α linolenic—ALA), C20:2—eicosadienoic acid, C20:3—eicosatrienoic acid, C20:4—ETA, C20:5—EPA, C22:6—DHA.

PUFA abundance (as % of total FA) of the three strains increased following the exposure to warming alone or in combination with acidification. However, each strain showed a specific response in PUFA composition (Fig. 3), especially in the relative abundance of individual FA (Table S2a,b). Numerical data and the relative change in abundance are available in Supplementary S2.

### Omega-3 and omega-6 FA

One of our study’s hypotheses was that long-term exposure to warming and higher CO_2_ alters the profile of omega-3 and omega-6 FA, decreasing the n-3/n-6 ratio. The focus was mainly on omega-3 PUFA: DHA, EPA, ALA and omega-6 PUFA: LA, ARA. When present, eicosatetraenoic acid (omega-3, ETA) and *trans*-Linoleic acid (omega-6, trans LA) were also analyzed to provide further insights into essential FA synthesis.

#### Omega-3 profiles (n-3)

**Fig. 4:**
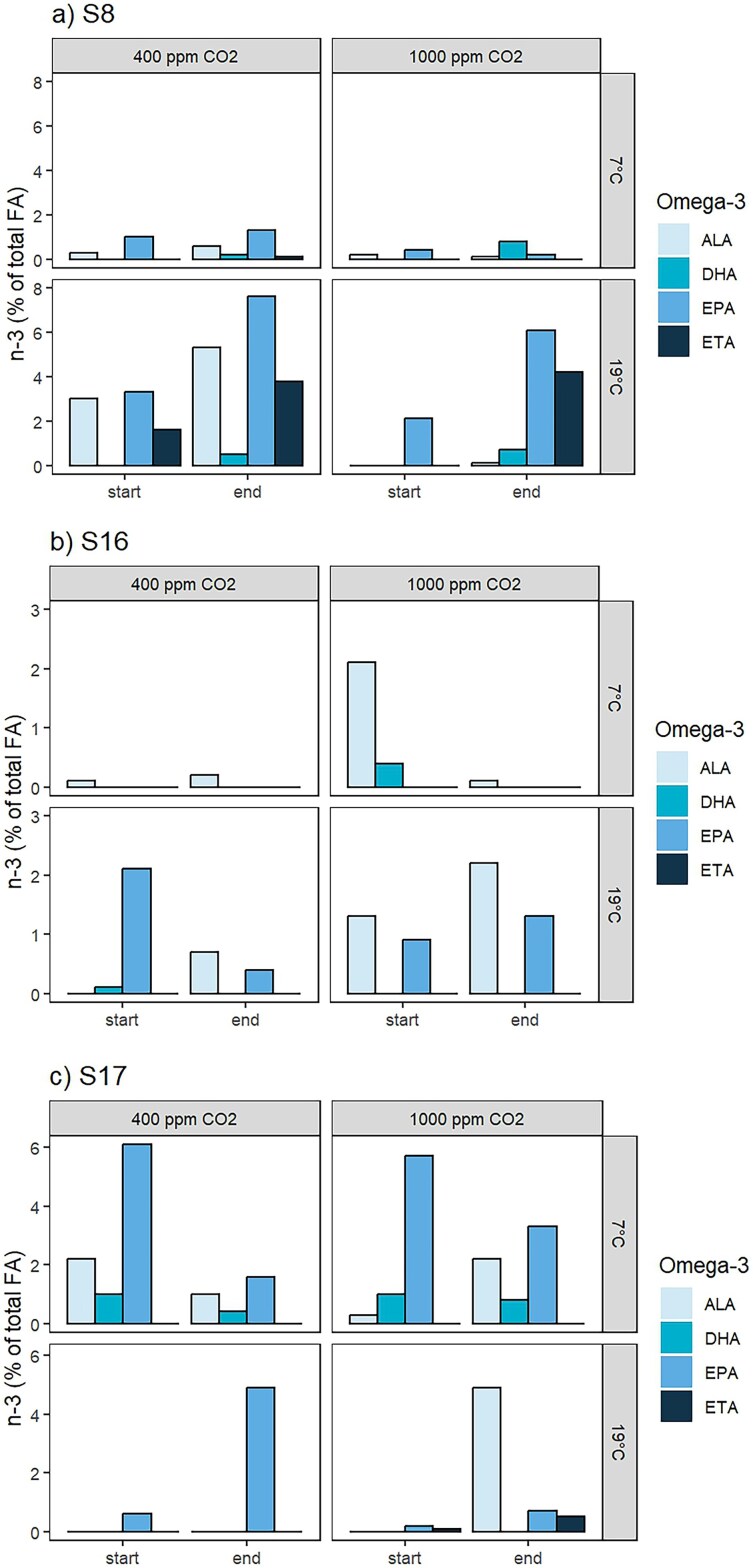
Omega-3 FA found in strains a) S8, b) S16 and c) S17 after the long-term exposure. Values on Y axis represent the relative abundance (% of total FA) for all omega-3 FA (sum).

For strain S8, the most abundant representative of omega-3 FA was EPA, with total abundance ranging from 0 to 8% of total FA. EPA abundance was highest in warming conditions (higher EPA at 19°C compared to 7°C), increasing by the end of the exposure for the ambient CO_2_ (19°C × 400 ppm) and high CO_2_ conditions (19°C × 1000 ppm, Fig. 4a).

S16 and S17 displayed different patterns in EPA production under the influence of individual drivers—warming or acidification. Specifically, EPA was not identified in any CO_2_ scenario for S16 in low temperatures (7°C, Fig. 4b). For S17, however, the exposure to both CO_2_ scenarios (400 and 1 000 ppm) led to a decrease in EPA abundance in the cold temperature regime (7°C). Moreover, the divergent responses of S16 and S17 in EPA production were highlighted in the warming scenario, where S16 produced less EPA after the long-term exposure to warming (Fig. 4b,c). In contrast, EPA abundance of S17 increased nearly 10× after the exposure to warming. However, both S16 and S17 exhibited more EPA% under the combined effects of warming and acidification than in the beginning.

A similar trend was observed for ETA (a homolog of EPA with four double bonds), where the combined effect of the two drivers led to the highest increase in ETA abundance for two of the strains: S8 and S17 (19°C × 1000 ppm, Fig. 4a,c). ETA was not detected in *S. marinoi* strain S16 in any of the scenarios, yet it is uncertain whether it was not produced or was further converted to EPA (Supplementary S4).

ALA abundance was similar to EPA in S8, ranging from 0 to 5% (of total FA). It was considerably more abundant under ambient CO_2_ (400 ppm) than under higher levels (1 000 ppm). There was also a higher percentage in warmer temperatures (19 vs 7°C, Fig. 4a). Under the combined effect of warming and acidification, both S16 and S17 responded with an increase in ALA abundance after exposure. The individual action of warming led to higher ALA abundance only in S8 and S16 (Fig. 4a,b). In contrast, for S17, acidification alone strongly enhanced ALA production, with levels up to seven times higher than at the start (Fig. 4c).

DHA was considerably less abundant than EPA, ranging from 0 to 1% of total FA. Among the omega-3 essential FA investigated, DHA was the most heterogenous and strain-specific. In northern strain S8, DHA production increased in all tested scenarios (Fig. 4a), being most abundant in high CO_2_ conditions (1000 ppm). In contrast, DHA was not detected in strains S16 and S17 after long-term exposure to both drivers (19°C × 1000 ppm). Additionally, acidification alone reduced DHA levels in both S16 and S17 (Fig. 4b,c), while warming alone led to a decrease in DHA only in S16.

### Omega-6 profiles (n-6)

**Fig. 5:**
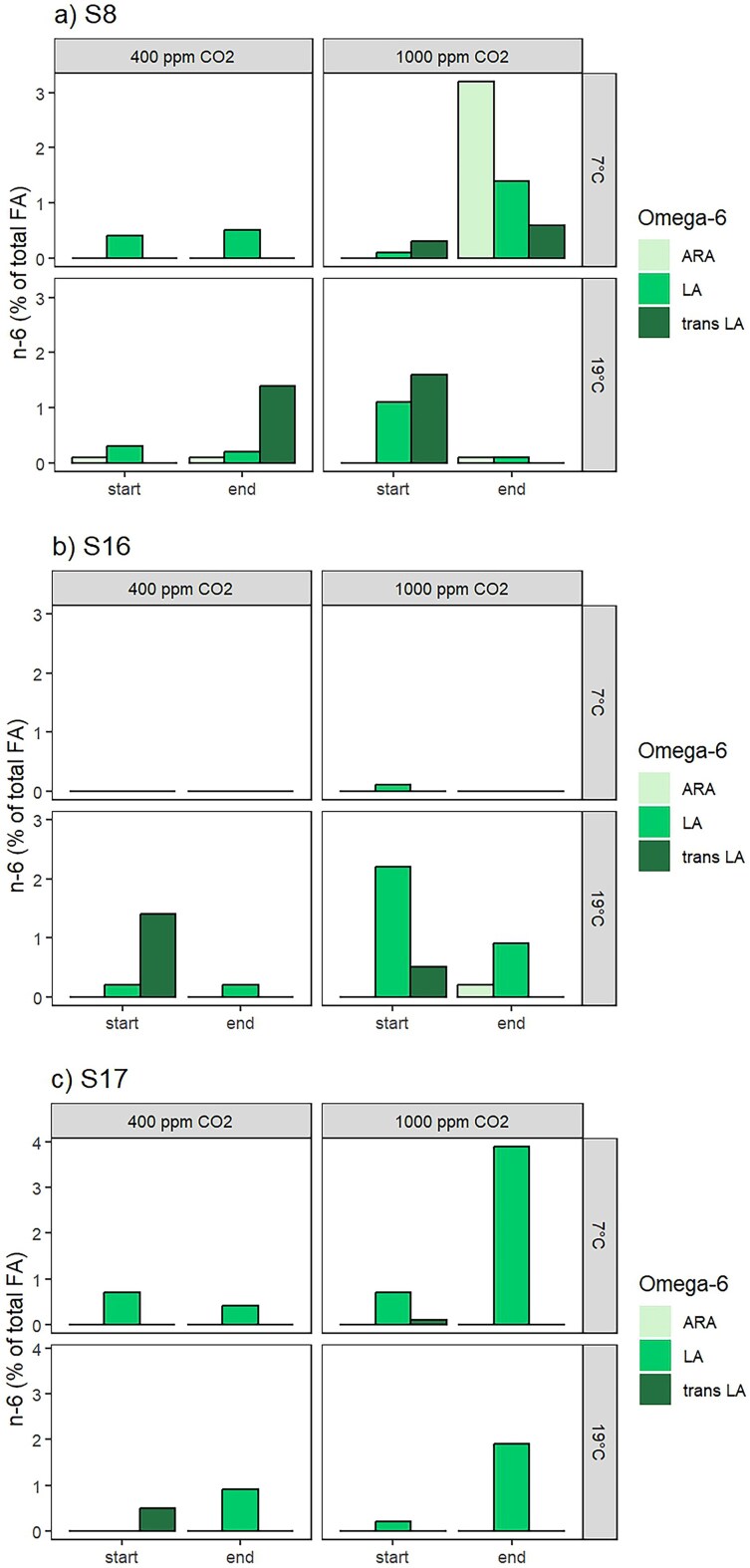
Omega-6 FA found in strains a) S8, b) S16 and c) S17 after the long-term exposure. Values on Y axis represent the relative abundance (% of total FA) of all omega-6 FA (sum).

For strain S8, LA was positively influenced by acidification alone, showing higher abundance under high CO_2_ (Fig. 5a). Warming led to less LA% in S8 for both CO_2_ levels, yet the impact was more evident for the combined scenario (19°C × 1000 ppm). S16 and S17 responded differently to the influence of warming and acidification in terms of LA production. Specifically, by acting alone or combined, the two drivers decreased LA abundance of S16 and increased it for S17 by the end of the experiment (Fig. 5b,c). No LA was identified in S16 under the ambient scenario (7°C × 400 ppm). Trans LA was identified in most treatments (except under ambient conditions), with a general decreasing trend toward the end of the experiment, except in S8 (high CO₂ and high temperature assays).

The abundance of ARA was generally enhanced by acidification, either alone (in strain S8, Fig. 5a) or in combination with warming (in strains S8 and S16; Fig. 5a,b). Warming alone led to a slight decrease in ARA abundance in S8, while in S17, ARA was not detected under any of the experimental scenarios.

### Omega-3: omega-6 ratio (n-3/n-6)

**Fig. 6:**
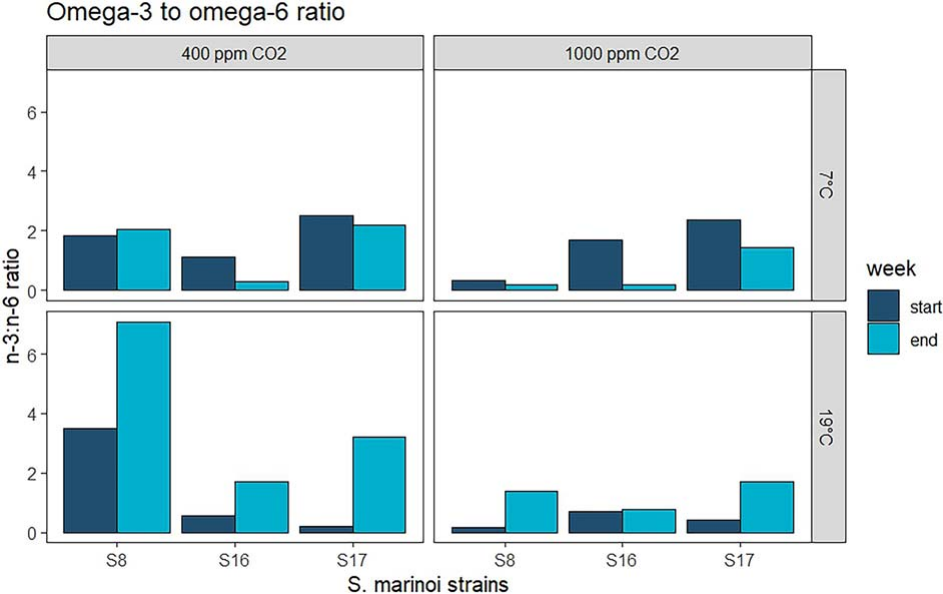
Changes in the omega-3 to omega-6 ratio (n-3/n-6) after the long-term exposure to ambient (7°C × 400 ppm), warming (19°C × 400 ppm), acidification (7°C × 1000 ppm) or coupled (19°C × 1000 ppm) scenarios. Values on Y axis represent the n-3/n-6 ratio. n-3 and n-6 were calculated by summing up all omega-3 and omega-6 FA respectively.

The n-3/n-6 ratio increased after exposure of strain S8 to ambient conditions (end vs. start, 7°C × 400 ppm) as well as after adaptation to warming alone (19°C × 400 ppm) or combined with acidification (19°C × 1000 ppm; Fig. 6). In contrast, the n-3/n-6 ratio of S8 significantly decreased under acidification alone (7°C × 1000 ppm; Kruskal-Wallis, *P* = 0.02). Although not statistically significant, S16 and S17 exhibited higher n-3/n-6 ratios at the end of the exposure to warm as compared to cold temperatures (19 vs 7°C, *P* = 0.2). However, in all three strains, the n-3/n-6 ratio significantly increased after exposure to warming and acidification combined (19°C × 1000 ppm; Kruskal-Wallis, *P* = 0.049). Acidification appeared to be the main driver of decreased n-3/n-6 values (*P* = 0.03).

## DISCUSSION

All three *S. marinoi* strains displayed divergent trends in PUFA synthesis for the warming × acidification scenarios, underscoring the importance of considering multiple phytoplankton strains and populations when investigating possible responses to climate change. Warming, acting alone or combined with acidification, did not decrease PUFA levels in any of the strains. Consistent with (Wu *et al.*, 2022), acidification alone increased PUFA% in S8, but in our case, it also led to a reduction in PUFA% in S16, with minimal impact on S17. In contrast, both the individual and combined effects of warming and elevated CO_2_ resulted in higher PUFA levels in S8, S16 and S17. These strain-specific patterns in PUFA synthesis are ecologically relevant, as they directly influence the nutritional quality of diatoms for their grazers. *Skeletonema* sp. can be an important food source for copepods (which are one of the most abundant and diverse groups of zooplankton in oceans and a key component of aquatic food webs), through influencing their egg lipid composition and hatching success (Koski *et al.*, 2012). Therefore, understanding how climatic change impacts the quality of organisms such as *Skeletonema* sp., will have implications for higher trophic levels.

The presence or absence of certain PUFA in our *S. marinoi* strains (in several experimental conditions: EPA, DHA; see Fig. 4, Table S2a) could be explained by their underlying metabolic pathways (Supplementary S4), as some FA serve as precursors for others (e.g. ALA is a precursor for EPA and DHA, while LA is a precursor for ARA) (Monroig and Kabeya, 2018). In a phenomic study, Johansson *et al.*, (2019) found that a mutant strain of *S. marinoi*, which was more tolerant to increased temperatures than the wild type, contained a specific FA transport protein, which plays a crucial role in thermal adaptation and FA production. Loss of the protein was associated with relative accumulation of EPA and reduced DHA synthesis, especially at higher temperatures (24°C). In our case, lower DHA levels were generally associated with higher EPA levels, which might reflect a cost-efficient metabolic strategy.

It has been well documented that exposure to acidification or warming leads to increased abundance of DHA (Leu *et al.*, 2013; Wang *et al.*, 2017; Fu *et al.*, 2023). Complementing these studies, our results showed that the combined action of acidification and warming also led to higher DHA levels in strain S8. Interestingly, this pattern did not apply to S16 and S17, where exposure to warming and acidification—either separately or in combination—led to a decrease or complete inhibition of DHA synthesis. Shang *et al.*, (2024) also found that warming alone decreased PUFA levels in *Skeletonema dohrnii*, especially for DHA and EPA. ALA is the precursor of some FA with longer chains (e.g. EPA, DHA) and in our strains, S16 and S17, both ALA and EPA increased after exposure to the warming x CO_2_ scenario. We cannot exclude that ALA might have been converted to EPA rather than DHA, thus explaining the lower DHA abundance in the two strains. The further conversion of EPA to DHA requires several enzymatic steps (Supplementary S4, Monroig and Kabeya, 2018) all of which imply a higher energetic cost and could explain the reduced DHA in the warming x CO_2_ scenario. Notably, even though S16 and S17 originated from the same location, they differed in their ALA responses to warming and acidification, underscoring the substantial intraspecific variation and physiological plasticity within *S. marinoi*.

However, a meta-analysis conducted by (Jónasdóttir, 2019) and a screening of 235 microalgal strains (Mitani et al., 2017) revealed diatoms as the main suppliers of EPA, while DHA is preferentially produced by dinoflagellates and coccolitophores (*Emiliania huxleyi*), with amounts between 1 and 3% being synthesized by diatoms (Remize et al., 2022). Our data suggests that strains like S8 might be able to increase DHA synthesis from 0 to 0.8% as a response to warming alone or coupled with acidification. The combined action of warming and acidification also led to a decrease in LA levels in two of the studied strains (S8 and S16), while for strain S17, LA levels increased after exposure to both stressors. Lower LA levels could also result in reduced synthesis of other omega-6 FA, such as ARA or trans LA, due to the LA’s role as a precursor in their synthesis (Supplementary S4, Hanna and Hafez, 2018). This relationship was highlighted in strains S8 and S16, where trans LA was also detected, but absent in S17. The distinct responses of the three *S. marinoi* strains to the individual action of warming and acidification in terms FA production might affect the nutritional quality of phytoplankton as a food source, with potential implications for both aquatic consumers and human health (Hirata *et al.*, 2020; Guo *et al.*, 2023).

As primary producers, phytoplankton—especially diatoms—play a central role in providing FA to higher trophic levels, acting as the main producers of unsaturated FA in marine ecosystems. Here, we showed that, although this is a strain-specific feature, the concentration of unsaturated FA in *S. marinoi* generally increases under elevated temperature, as well as under high temperature when combined with OA conditions. A similar trend under elevated temperatures was observed by Teoh *et al.* (2004) and more recently in the marine diatom *Thalassiosira pseudonana*, which produced twice as much omega-3 FA in strains adapted for 2.5 years to high temperatures compared to cold-adapted ones (O’Donnell *et al.*, 2019). This was unexpected, as we hypothesized that warming—or its combined effect with acidification—would decrease the n-3/n-6 ratio. These results are therefore opposite to those supporting the homeoviscous adaptation theory, which is largely based on short-term observations (Jiang and Gao, 2004; Guschina and Harwood, 2006). Our results suggest that, over longer (evolutionary) timescales, marine diatoms can adapt and adjust unsaturated FA synthesis, which might otherwise be reduced in the short term. One consequence associated with the effects of these two stressors on aquatic ecosystems is the increase of omega-6 abundance (Tan and Zheng, 2022; Tan *et al.*, 2024, 2025), which causes a drop in the n-3/n-6 ratio and is considered an indicator for lower food quality for zooplankton (Taipale *et al.*, 2018) and indirectly for humans. Alterations in the FA composition in phytoplankton could propagate through the trophic network, potentially affecting human diets (Puccinelli *et al.*, 2021), with negative consequences such as cardiovascular risk, chronic inflammation, and obesity (Russo, 2009; Tortosa-Caparrós *et al.*, 2017). Although omega-6 FA offer physiological benefits—such as reducing low-density lipoprotein (LDL) cholesterol—maintaining a balance between omega-6 and omega-3 is essential. In marine fish, the omega-6 content is relatively low, with the values of n-3/n-6 varying from 5 to 10 (Valfré *et al.*, 2003). Therefore, a higher n-3/n-6 ratio is desirable for high-quality food sources, consistent with our results when *S. marinoi* was exposed to both stressors in combination. While these findings suggest that global change may not necessarily reduce the lipid content of marine phytoplankton, further studies are needed to directly assess how such changes translate to effects in marine ecosystems.

## CONCLUSIONS

This study provides evidence that long-term exposure to elevated temperature and CO₂ can induce distinct and strain-specific changes in the FA composition in the marine diatom *S. marinoi*. While PUFA levels were variably affected across strains, omega-3 FA generally increased under warming, especially when combined with acidification. In contrast, acidification alone decreased the omega-3 to omega-6 ratio, potentially reducing the nutritional quality of phytoplankton for higher trophic levels. However, the combination of increased CO_2_ and temperature displayed the opposite trend. Our results suggest that, in the context of climate change, diatoms—key contributors to lipid production in aquatic food webs could alter their FA composition, thereby affecting the dietary intake of organisms at higher trophic levels. The diverse responses observed among the three strains highlight the metabolic plasticity of *S. marinoi* and the importance of accounting for intraspecific variability when predicting phytoplankton responses to global change. These findings underscore the need to consider both individual and combined effects of climate-related stressors to better assess their ecological consequences, particularly in terms of food web structure and functioning.

## Supplementary Material

4_PUFA_Resubmission_Supplementary_fbag057

## Data Availability

The data is available from the corresponding author on request.
