# Supplementary material for: Warming and acidification alter essential fatty acid profiles in marine diatom Skeletonema marinoi
Source: J Plankton Res. 2026 Jul 3;48(4):fbag057. doi: 10.1093/plankt/fbag057 (PMC13330538; doi:10.1093/plankt/fbag057)

Supplementary material for **Warming and acidification alter essential fatty acid profiles in marine diatom *Skeletonema marinoi***

Maria Nicoară^1,2*^, Charlotte L. Briddon^1,3*^, Alexandra Mortu^4^, Adriana Hegedűs^1^, Emese Gal^5^, Horia Leonard Banciu^6,7^, Bogdan Drugă^1
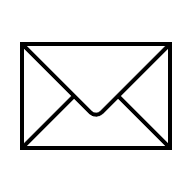
^

^1^ Institute of Biological Research (NIRDBS), 48 Republicii Street, 400015, Cluj-Napoca, Romania

^2^ Doctoral School on Integrative Biology, Faculty of Biology and Geology, Babeș-Bolyai University, Cluj-Napoca, Romania

^3^ School of Geographical Sciences, University of Bristol, University Road, Bristol BS8 1SS, United Kingdom

^4^ Faculty of Life Sciences, University of Vienna, 1 Djesrassiplatz, 1030 Vienna, Austria

^5^ Department of Chemistry, Babeș-Bolyai University, 11 Arany Janos, Cluj-Napoca 400028, Romania

^6^ Department of Molecular Biology and Biotechnology, Faculty of Biology and Geology, Babeș-Bolyai University, Cluj-Napoca, Romania

^7^Centre for Systems Biology, Biodiversity and Bioresources, Faculty of Biology and Geology, Babeș-Bolyai University, Cluj-Napoca, Romania.

* These authors contributed equally to this work

**S1. Qualitative assessment of fatty acid (FA) types identified in the *S. marinoi* strains S8, S16 and S17. Values represent the number of observations and the relative abundance of saturated (SFA), monounsaturated (MUFA) and polyunsaturated (PUFA) to the total FA in each strain.** More than 90% of the compounds identified in all strains using FAME (Fatty Acid Methyl Esters) consisted of FA, with the remaining fraction attributed to other compounds (“others”) or unidentified (”na”). Specifically, at 1000 ppm, in addition to fatty acids, the cold-adapted S8 exhibited, compounds such as vitamin E (0.63% of total lipids), cholesterol (1.87%), and citric, benzene acetic and acetylenic acids (0.08%, 0.01% and 0.22% of total lipids, respectively). Citric acid was also detected in cold-adapted S8 at 400 ppm (0.02%). No compounds other than fatty acids were identified in the FAME chromatograms of *S. marinoi* strains S16 or S17.

|  | **Skeletonema marinoi strains** | | | | | |
| --- | --- | --- | --- | --- | --- | --- |
|  | **S8** | | **S16** | | **S17** | |
| **FA type** | **7°C** | **19°C** | **7°C** | **19°C** | **7°C** | **19°C** |
| SFA | 88 (27%) | 56 (17%) | 55 (22%) | 58 (22%) | 52 (21%) | 46 (19%) |
| MUFA | 39 (12%) | 36 (11%) | 24 (9%) | 34 (13%) | 29 (12%) | 30 (12%) |
| PUFA | 56 (17%) | 50 (15%) | 35 (14%) | 48 (19%) | 45 (19%) | 40 (17%) |
| Total FA | 183 (56%) | 142 (43%) | 114 (45%) | 140 (55%) | 126 (52%) | 116 (48%) |

**S2a. PUFA abundance after the long-term exposure to warming, acidification and both combined.** The experiment followed a 2x2 factorial design – 2 factors: temperature and CO_2_, with 2 levels each (7ºC, 19ºC and 400 ppm, 1000 ppm). Values represent PUFA abundance ( % of total fatty acids).

|  |  |  |  | PUFA% | | | | | | | | | |
| --- | --- | --- | --- | --- | --- | --- | --- | --- | --- | --- | --- | --- | --- |
| strain | CO_2_ | t(ºC) | timepoint | C16:2 | C16:3 | C17:3 | C18:2 | C18:3 | C20:2 | C20:3 | C20:4 | C20:5 | C22:6 |
| S8 | 400 ppm | 7ºC | start | 3.43 | 0.07 | 0 | 2.73 | 0.32 | 0.05 | 0 | 0 | 0.99 | 0 |
|  |  |  | end | 0 | 0 | 0 | 0.52 | 0.64 | 0 | 0.11 | 0.1 | 1.26 | 0.22 |
|  |  | 19ºC | start | 1.19 | 0 | 0 | 1.27 | 3.06 | 0 | 0 | 1.79 | 3.32 | 0.17 |
|  |  |  | end | 0.73 | 0.22 | 0 | 1.65 | 5.42 | 0 | 0.16 | 4.02 | 7.8 | 0.51 |
|  | 1000 ppm | 7ºC | start | 2.41 | 0.15 | 0.03 | 0.63 | 0.23 | 0 | 0.04 | 0 | 0.48 | 0 |
|  |  |  | end | 0.6 | 1.41 | 0.18 | 2.9 | 0.05 | 0 | 0 | 3.21 | 0.17 | 0.83 |
|  |  | 19ºC | start | 8.77 | 0 | 1.15 | 2.76 | 0 | 0 | 0 | 0 | 2.16 | 0 |
|  |  |  | end | 0.03 | 8.14 | 0 | 1.93 | 0.15 | 0 | 0.14 | 4.49 | 6.36 | 0.69 |
| S16 | 400 ppm | 7ºC | start | 2.38 | 0 | 0.22 | 0.11 | 0.36 | 0 | 0.2 | 0 | 0 | 0 |
|  |  |  | end | 3.62 | 0 | 0.2 | 0.63 | 0.13 | 0 | 0.13 | 0 | 0 | 0 |
|  |  | 19ºC | start | 1.6 | 0.12 | 0 | 2.49 | 0 | 0 | 0 | 0 | 2.11 | 0.12 |
|  |  |  | end | 3.37 | 0.16 | 0.21 | 1.69 | 0.67 | 0 | 0 | 0 | 0.35 | 0 |
|  | 1000 ppm | 7ºC | start | 0.85 | 0.1 | 0 | 1.51 | 2.07 | 0 | 0.66 | 0 | 0 | 0.43 |
|  |  |  | end | 3.84 | 0.04 | 0.58 | 0.35 | 0.13 | 0 | 0 | 0 | 0 | 0 |
|  |  | 19ºC | start | 0.7 | 0 | 0 | 3.27 | 1.26 | 0 | 0.04 | 0 | 0.9 | 0.04 |
|  |  |  | end | 2.71 | 0.1 | 1 | 1.23 | 2.19 | 0 | 0 | 0.37 | 1.06 | 0 |
| S17 | 400 ppm | 7ºC | start | 0.91 | 0.09 | 2.62 | 0.73 | 2.2 | 0 | 0.3 | 0 | 6.05 | 0.96 |
|  |  |  | end | 1.66 | 0 | 0.96 | 0.42 | 1.04 | 0 | 0 | 0 | 1.62 | 0.38 |
|  |  | 19ºC | start | 2.29 | 0 | 0 | 1.01 | 0 | 0 | 0 | 0 | 0.56 | 0 |
|  |  |  | end | 0.73 | 0.09 | 0.17 | 0.99 | 0 | 0 | 0.02 | 0 | 5.53 | 0 |
|  | 1000 ppm | 7ºC | start | 0.65 | 0 | 0 | 3.08 | 0.3 | 0 | 0.76 | 0.01 | 5.74 | 0.97 |
|  |  |  | end | 1.14 | 0.12 | 0 | 3.99 | 2.21 | 0 | 0 | 0 | 3.34 | 0.76 |
|  |  | 19ºC | start | 4.08 | 0.1 | 0.02 | 0.57 | 0 | 0 | 0 | 0.07 | 0.15 | 0 |
|  |  |  | end | 1.46 | 0 | 0.34 | 1.88 | 4.94 | 0 | 0.15 | 0.45 | 0.72 | 0 |

**S2b PUFA relative change after the long-term exposure to warming, acidification and both combined.** Relative change was calculated based on the following formula (End - Start)/ Start *100, where End = Final abundance and Start = Initial abundance of each PUFA.

The relative change was considered 0 where PUFA were absent in both beginning and end of experiment. If no PUFA was detected at the beginning, the initial abundance was considered 0.01% (due to the impossibility of dividing to 0), resulting in relative changes > +1000%. If no PUFA was found at the end, the final abundance was considered as 0% and the relative change was considered -100%.

|  | | | PUFA relative change (%) | | | | | | | | | |
| --- | --- | --- | --- | --- | --- | --- | --- | --- | --- | --- | --- | --- |
| strain | CO2 | t(ºC) | C16:2 | C16:3 | C17:3 | C18:2 | C18:3 | C20:2 | C20:3 | C20:4 | C20:5 | C22:6 |
| S8 | 400 ppm | 7ºC | -100 | -100 | 0 | -80.9524 | 100 | -100 | 1000 | 900 | 27.27273 | 2100 |
|  |  | 19ºC | -38.6555 | 2100 | 0 | 29.92126 | 77.12418 | 0 | 1500 | 124.581 | 134.9398 | 200 |
|  | 1000 ppm | 7ºC | -75.1037 | 840 | 500 | 360.3175 | -78.2609 | 0 | -100 | 32000 | -64.5833 | 8200 |
|  |  | 19ºC | -99.6579 | 81300 | -100 | -30.0725 | 1400 | 0 | 1300 | 44800 | 194.4444 | 6800 |
| S16 | 400 ppm | 7ºC | 52.10084 | 0 | -9.09091 | 472.7273 | -63.8889 | 0 | -35 | 0 | 0 | 0 |
|  |  | 19ºC | 110.625 | 33.33333 | 2000 | -32.1285 | 6600 | 0 | 0 | 0 | -83.4123 | -100 |
|  | 1000 ppm | 7ºC | 351.7647 | -60 | 5700 | -76.8212 | -93.7198 | 0 | -100 | 0 | 0 | -100 |
|  |  | 19ºC | 287.1429 | 900 | 9900 | -62.3853 | 73.80952 | 0 | -100 | 3600 | 17.77778 | -100 |
| S17 | 400 ppm | 7ºC | 82.41758 | -100 | -63.3588 | -42.4658 | -52.7273 | 0 | -100 | 0 | -73.2231 | -60.4167 |
|  |  | 19ºC | -68.1223 | 800 | 1600 | -1.9802 | 0 | 0 | 100 | 0 | 887.5 | 0 |
|  | 1000 ppm | 7ºC | 75.38462 | 1100 | 0 | 29.54545 | 636.6667 | 0 | -100 | -100 | -41.8118 | -21.6495 |
|  |  | 19ºC | -64.2157 | -100 | 1600 | 229.8246 | 49300 | 0 | 1400 | 542.8571 | 380 | 0 |

**S3. Linear models, AIC selection and post-hoc analysis results** showing the change in the omega-3 to omega-6 ratio (used as an indicator for food quality) as a function of temperature (temp), CO_2_ and long-term adaptation to stressors (week). Relates to Figure 6. Values of P < 0.05 were considered significant (in bold), while P = 0.06 were considered borderline significant (underlined). CL = 95% confidence level. Interactions were considered and removed if p > 0.25. Based on AIC values, model 2 fitted best.

| model 1  lm(log(ratio36) ~ temp+co_2_+week | Df | Sum Sq | F value | Pr (>F) |
| --- | --- | --- | --- | --- |
| Temperature | 1 | 0.0466 | 0.0430 | 0.83773 |
| CO_2_ | 1 | 4.0577 | 3.7487 | 0.06711 |
| Week | 1 | 0.5182 | 0.4788 | 0.49693 |
| Residuals | 20 | 21.6481 |  |  |

| model 2  lm(log(ratio36) ~ strain*co_2_+week | Df | Sum Sq | F value | Pr (>F) |
| --- | --- | --- | --- | --- |
| Strain | 2 | 1.7508 | 1.0240 | 0.38029 |
| CO_2_ | 1 | 4.0577 | 4.7463 | **0.04372** |
| Week | 1 | 0.5182 | 0.6062 | 0.44693 |
| Strain:CO_2_ | 2 | 5.4103 | 3.1643 | 0.06789 |
| Residuals | 17 | 14.5335 |  |  |

| model 3  lm(log(ratio36) ~ co_2_*week*temp | Df | Sum Sq | F value | Pr (>F) | |
| --- | --- | --- | --- | --- | --- |
| CO_2_ | 1 | 4.0577 | 4.5265 | | **0.04927** |
| Week | 1 | 0.5182 | 0.5781 | | 0.45811 |
| Temperature | 1 | 0.0466 | 0.0520 | | 0.82254 |
| CO_2_:Week | 1 | 0.3407 | 0.3800 | | 0.54625 |
| CO_2_:Temperature | 1 | 0.0144 | 0.0161 | | 0.90069 |
| Week:Temperature | 1 | 6.9008 | 7.6981 | | **0.01353** |
| CO_2_:Week:Temperature | 1 | 0.0495 | 0.0552 | | 0.81728 |
| Residuals | 16 | 14.342 | 0.8964 | |  |

AIC model selection

|  | Df | AIC |  |
| --- | --- | --- | --- |
| model 1 | 5 | 75.6337 |  |
| model 2 | 8 | **72.0707** |  |
| model 3 | 9 | 73.7536 |  |

Post-hoc analysis for strain*CO_2_ interaction from model 2

| S8:CO2 | emmean | SE | Df | Lower CL | Upper CL | P value |
| --- | --- | --- | --- | --- | --- | --- |
| 400 ppm CO_2_ | 1.132 | 0.462 | 17 | 0.156 | 2.1072 | **0.0041** |
| 1000 ppm CO_2_ | -1.033 | 0.462 | 17 | -2.008 | -0.0573 |  |
|  |  | | | | | |
| **S16:CO2** | **emmean** | **SE** | **Df** | **Lower CL** | **Upper CL** | **P value** |
| 400 ppm CO_2_ | -0.283 | -0.462 | 17 | -1.259 | 0.6919 | **0.7752** |
| 1000 ppm CO_2_ | -0.473 | -0.462 | 17 | -1.449 | 0.5022 |  |
|  |  | | | | | |
| **S17:CO2** | **emmean** | **SE** | **Df** | **Lower CL** | **Upper CL** | **P value** |
| 400 ppm CO_2_ | 0.329 | 0.462 | 17 | -0.646 | 1.3044 | **0.8650** |
| 1000 ppm CO_2_ | 0.216 | 0.462 | 17 | -0.759 | 1.1916 |  |

The plot for model 2 shows significant differences between the CO_2_ treatments in strain S8.
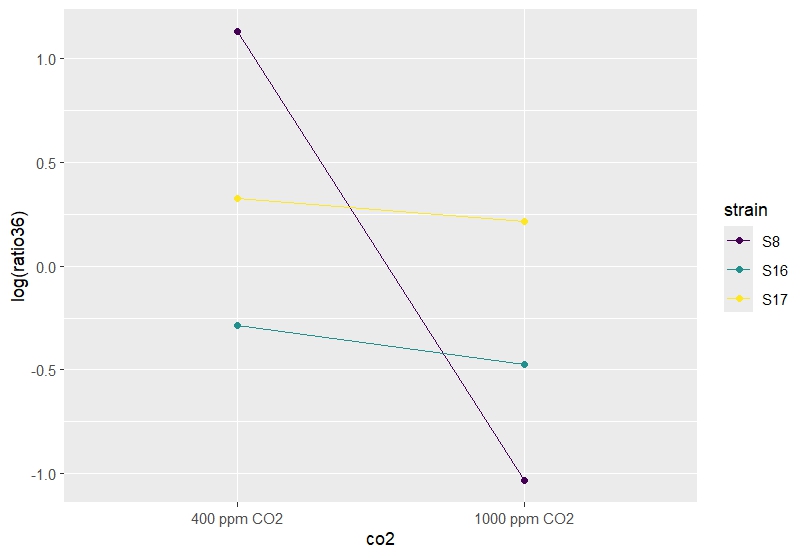


**S4. Schematic representation of PUFA synthesis (adapted from Venegas-Caleron, 2010)**

Venegas-Calerón, M., Sayanova, O., Napier, J.A. (2010) An alternative to fish oils: Metabolic engineering of oil-seed crops to produce omega-3 long chain polyunsaturated fatty acids. *Prog Lipid Res*. *49*(2):108-19. doi: 10.1016/j.plipres.2009.10.001.


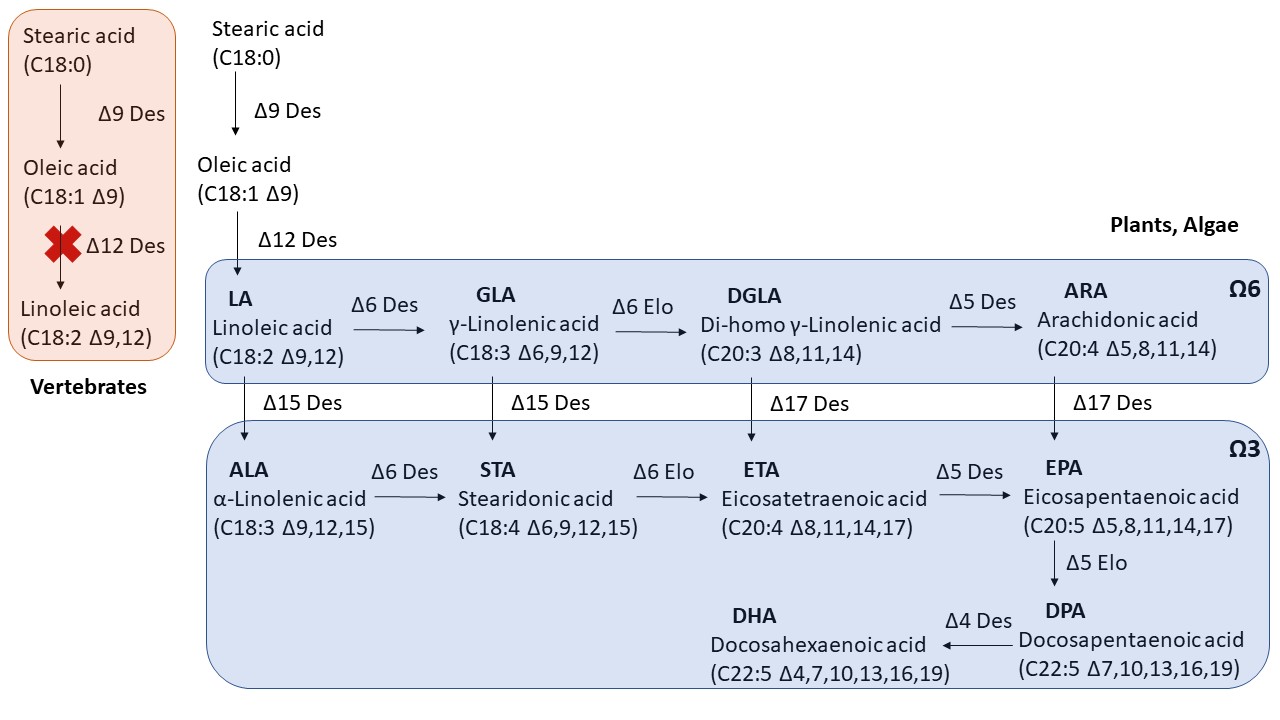

Supplement: 4_PUFA_Resubmission_Supplementary_fbag057 [file 4_pufa_resubmission_supplementary_fbag057.docx]
